# Sirolimus alters lung pathology and viral load following influenza A virus infection

**DOI:** 10.1186/s12931-017-0618-6

**Published:** 2017-07-11

**Authors:** Ahmed R. Alsuwaidi, Junu A. George, Saeeda Almarzooqi, Stacey M. Hartwig, Steven M. Varga, Abdul-Kader Souid

**Affiliations:** 1 Department of Pediatrics, College of Medicine and Health Sciences, United Arab Emirates University, Al Ain, United Arab Emirates; 2 Department of Pathology, College of Medicine and Health Sciences, United Arab Emirates University, Al Ain, United Arab Emirates; 3 Department of Microbiology & Immunology, Department of Pathology and Interdisciplinary Graduate Program in Immunology, University of Iowa, Iowa, USA

**Keywords:** Rapamycin, Influenza A virus, Viral replication, Inflammatory score, Lung function

## Abstract

**Background:**

Inhibitors of mTOR, such as sirolimus, have been shown to induce thymus involution and inflammatory lung disease in mice. The latter effect supports the role of this serine/threonine kinase in ameliorating lung inflammation. Other studies have shown sirolimus reduces/delays lung disease associated with various strains of influenza A virus (IAV). Thus, the effects of mTOR inhibitors on influenza infection deserve further studies.

**Methods:**

Here, we examined the changes in lung viral copies, pathology and pulmonary function associated with IAV (A/PR/8/34) infection in mice treated with sirolimus.

**Results:**

Body weight loss peaked between days 6–11 post-infection and was more severe in IAV-infected mice that were administered sirolimus as compared to mice that received IAV alone (*p* = 0.030). Natural log viral gene copies, mean ± SD per mg lung tissue, in IAV-infected mice that were administered sirolimus were 17.31 ± 1.27 on day 4, 19.31 ± 7.46 on day 10, and 0 on day 25. The corresponding number of copies in mice that received IAV alone were 18.56 ± 0.95 on day 4 (*p* = 0.132), 1.52 ± 1.39 on day 10 (*p* = 0.008), and 0 on day 25. Lung pathology was evident on days 4, 10, and 25 post infection, with mean ± SD inflammatory score of 9.0 ± 4.5 in IAV-infected mice that were administered sirolimus, as compared to 11.5 ± 4.5 (*p* = 0.335) in mice received IAV alone (maximum score, 26.0). Impaired lung function was evident in IAV-infected mice on days 4 and 10, as demonstrated by increased airway resistance and decreased compliance.

**Conclusions:**

In this model, the effects of sirolimus on influenza infection included severe weight loss and modified viral replication, respiratory function and lung inflammation. The adverse events associated with sirolimus treatment are consistent with its potent immunosuppressive activity and, thus, preclude its use in IAV infection.

## Background

Inhibitors of mTOR, such as, sirolimus, temsirolimus and everolimus, have been shown to induce inflammatory lung disease in mice [1]. This effect underscores the anti-inflammatory function of mTOR and supports its role in ameliorating lung inflammation [2]. The primary complex of mTOR mediates anti-inflammatory responses in the lung [2]. Sirolimus has also been shown to induce severe immunosuppression in mice, as a result of thymus involution due to cortical lymphocyte apoptosis [3].

In addition, inhibitors of mTOR are known to impair cellular bioenergetics [4, 5]. In one study, a prior brief exposure of Jurkat cells to sirolimus resulted in ~20% decrease in cellular respiration. This effect was accompanied by accumulation of cellular lactate and other biomarkers of anaerobic metabolism [4]. In another in vitro study, sirolimus administration decreased cellular respiration in several murine organs by 20–40% [5]. In contrast, the calcineurin inhibitors tacrolimus and cyclosporine exhibited no effects on mitochondrial respiration in the same tissues [5]. The impact of these metabolic derangements on the cytotoxicity of mTOR inhibitors, especially with respect to viral infections, has yet to be fully elucidated [6].

Influenza A virus (IAV) is a leading cause of lower respiratory tract infection that can result in severe lung disease [7]. The host immune response and virus-induced tissue damage both contribute to the severity of IAV-induced lung disease. At molecular level, IAV infection suppresses lung cellular respiration [8].

The combination of sirolimus and corticosteroid was reported to improve the outcome of patients with severe H1N1-induced pneumonia [9, 10]. However, the underlying mechanism to account for the therapeutic benefit of inhibition of mTOR activity during IAV infection remains unclear. A recent study has shown that sirolimus treatment during H3N2 virus infection protected mice against lethal secondary H5N1 virus infection [11]. Transfer of serum was sufficient to protect naïve mice against H5N1 infection. This enhanced protection mediated by the sirolimus treated mice was attributed to altered B cell class switching and the induction of effective cross-reactive antibodies [11]. Everolimus-treated mice exhibited reduced lung hemorrhage and lung weight in response to lethal H1N1 and H5N1 infections; the treatment delayed death but did not prevent mortality [12]. More recently, sirolimus was shown not to influence survival or antibody responses to influenza infections in mice [13].

Taken together, these data demonstrate that the role of mTOR in modulating influenza infection deserves further studies. Here, we sought to evaluate the impact of sirolimus treatment on lung inflammation, lung function and overall disease severity in mice following acute IAV infection.

## Methods

### Reagents

Sirolimus was purchased from MedChem Express, LLC (Princeton, NJ). The drug was dissolved in dimethyl sulfoxide (DMSO) at 50 μg/μL (55 mM) and stored in at −20 °C. This concentrated stock was diluted to a final concentration of 0.5 μg/μL in dH_2_O immediately before use. Ketamine (50 mg/mL), xylazine (suspended in methanol at 10 mg/mL), and remaining reagents were purchased from Sigma-Aldrich (St. Louis, MO).

Ketamine-xylazine solution consisted of 80 μL (4 mg) ketamine plus 20 μL (0.2 mg) xylazine and stored at 4 °C for a maximum of 2 weeks. The solution was diluted 1:10 with dH_2_O immediately prior to use. Mice were injected intraperitoneally with 10 μL/g (40 μg/g ketamine +2 μg/g xylazine) before inoculation and other surgical procedures. Adequate sedation was confirmed before intranasal inoculations by the absence of footpad reflexes. Mice were held in a supine position (at a 45° angle) with the back supported by the palm and the neck skin fold by the thumb and index finger. The inoculum was slowly released from a 10-μL micropipette as two small drops covering the two nostrils. The mice were allowed to inhale the volume without forming bubbles. They were then maintained in the same position until they regained consciousness and their rapid breathing returned to normal.

### Animals

BALB/c mice (4–8 weeks old; Jackson Laboratory, Bar Harbor, ME) were housed at 22 °C and 60% humidity. They had ad libitum access to standard rodent chow and filtered water. The experiments received approval from the Animal Ethics Committee-UAE University-College of Medicine and Health Sciences (Protocol No. A3–13).

### Intranasal inoculation and sirolimus treatment

Mice were anesthetized by the ketamine-xylazine mixture and intranasally inoculated (as conferred above) with 10^8.1^ TCID_50_ per nostril of IAV A/PR/8/34 (H1N1) on day 0 [8]. The mice were also injected intraperitoneally with either sirolimus [14] or an equal volume of the vehicle DMSO, both administered for 5 uninterrupted days every week for a total of 4 weeks beginning on the day of inoculation (day 0). Sirolimus solution was prepared immediately before use by diluting the 50 μg/μL original stock (in DMSO) with dH_2_O to a final concentration of 0.5 μg/μL. The sirolimus dose was 5 μg/g (10 μL/g). DMSO was also diluted 100-fold with dH_2_O and injected at 10 μL/g (actual DMSO dose, 0.1 μL/g).

### Tissue collection and processing

Specimens were collected as previously described [8]. For viral RNA extraction, one lung specimen was homogenized in TRIZOL (Invitrogen, Grand Island, NY) and the supernatant was stored at −80 °C. For histology, lung specimens were fixed in 4% phosphate-buffered paraformaldehyde and embedded in paraffin. Sections of the fixed tissue (5–7 μm thickness) were stained with hematoxylin and eosin (H&E) and examined under a light microscope.

A scoring system previously validated for respiratory syncytial virus infection was used to grade the intensity of disease [15, 16]. Histology sections were graded as follows: (A) degree of peribronchiolar/bronchial infiltrate (none, <25%, 25–75%, >75%); (B) type of peribronchiolar/bronchial infiltrate (none, interrupted collar, complete collar <5 cells thick, complete collar >5 cells thick); (C) bronchiolar/bronchial luminal exudate (none, ≤25% luminal occlusion, ≥25% luminal occlusion); (D) perivascular infiltrate (none, <10%, 10–50%, >50%); and (E) parenchymal pneumonia (none, patchy parenchymal infiltrate, heavy parenchymal infiltrate). The total score (ranged from 0 to 26) was set as: [A + 3(B + C) + D + E], Table 1.

**Table 1:** Scores of inflammation on Days 4, 10, and 25 post-infection^a^

	*DMSO*	*Sirolimus*	*IAV*	*Sirolimus + IAV*
	Day 4			
First mouse	0	3	-	-
Second mouse	-	-	13	7
Third mouse	-	-	13	8
	Day 10			
First mouse	3	4	11	4
Second mouse	-	-	3	18
Third mouse	-	-	15	12
	Day 25			
First mouse	3	3	7	7
Second mouse	3	3	13	7
Third mouse	3	3	17	b
Average (±SD) score	2.4 ± 1.3	3.2 ± 0.4	11.5 ± 4.5^*¥*^	9.0 ± 4.5^*¥*^

^*¥*^ *p* = 0.335

^a^Sections were scored as described in Methods (Cimolai et al., [15]; Hardy et al., [16])

^b^One mouse in the “sirolimus + IAV” group died on day 9

### Real-time (RT) PCR

Viral RNA was detected in the lung tissue of infected mice via RT-PCR [17]. Lungs were homogenized in 1.0 mL TRIZOL (Invitrogen, Carlsbad, CA), and cellular RNA was isolated from the supernatant by chloroform and isopropyl alcohol extraction. cDNA was prepared from two μg RNA using M-MLV Reverse Transcriptase (Promega, Madison, Wis.). Real-time PCR was performed to detect IAV *matrix* gene using previously described primers and probes [17], TaqMan® Universal PCR Master Mix and Applied Biosystems™ QuantStudio™ 7 Flex Real-Time PCR System (Foster City, CA). Amplification conditions were: an initial denaturation step at 94 °C for 5 min followed by 40 cycles of denaturation at 94 °C for 45 s and annealing at 60 °C for 1 min. Standard curve was prepared using cDNA from the virus sample (10^8.1^ TCID_50_).

### Evaluation of functional respiratory disease

Invasive pulmonary function analysis was performed using the FlexiVent instrument from SCIREQ (Montreal, PQ, Canada), as previously described [18]. This forced oscillation system measured respiratory disease in tracheotomized mice via changes in thoracic resistance (Rrs, cmH_2_O.s/mL), thoracic compliance (Crs, mL/cmH_2_O), large airway resistance (Rn, cmH_2_O.s/mL), lung tissue damping (resistance, G, cmH_2_O/mL), and lung tissue elastance (H, cmH_2_O/mL) at baseline and after methacholine challenges. Methacholine responsiveness was shown as area under the curve (AUC) of Rrs, Crs and Rn against methacholine concentration (0, 0.6, 1.25, 2.5, 5, and 10 mg/mL). Airway obstruction was also evaluated using the fast-flow maneuvers forced expiratory volume at 50 ms (FEV0.05, in mL).

### Statistical analysis

Data were analyzed using SPSS statistical package (version 20). The nonparametric test (2 independent variables; Mann–Whitney) was used to compare infected and uninfected samples. *P* < 0.05 was considered significant.

## Results

### Sirolimus augments the weight loss in IAV-infected mice

**Fig. 1:**
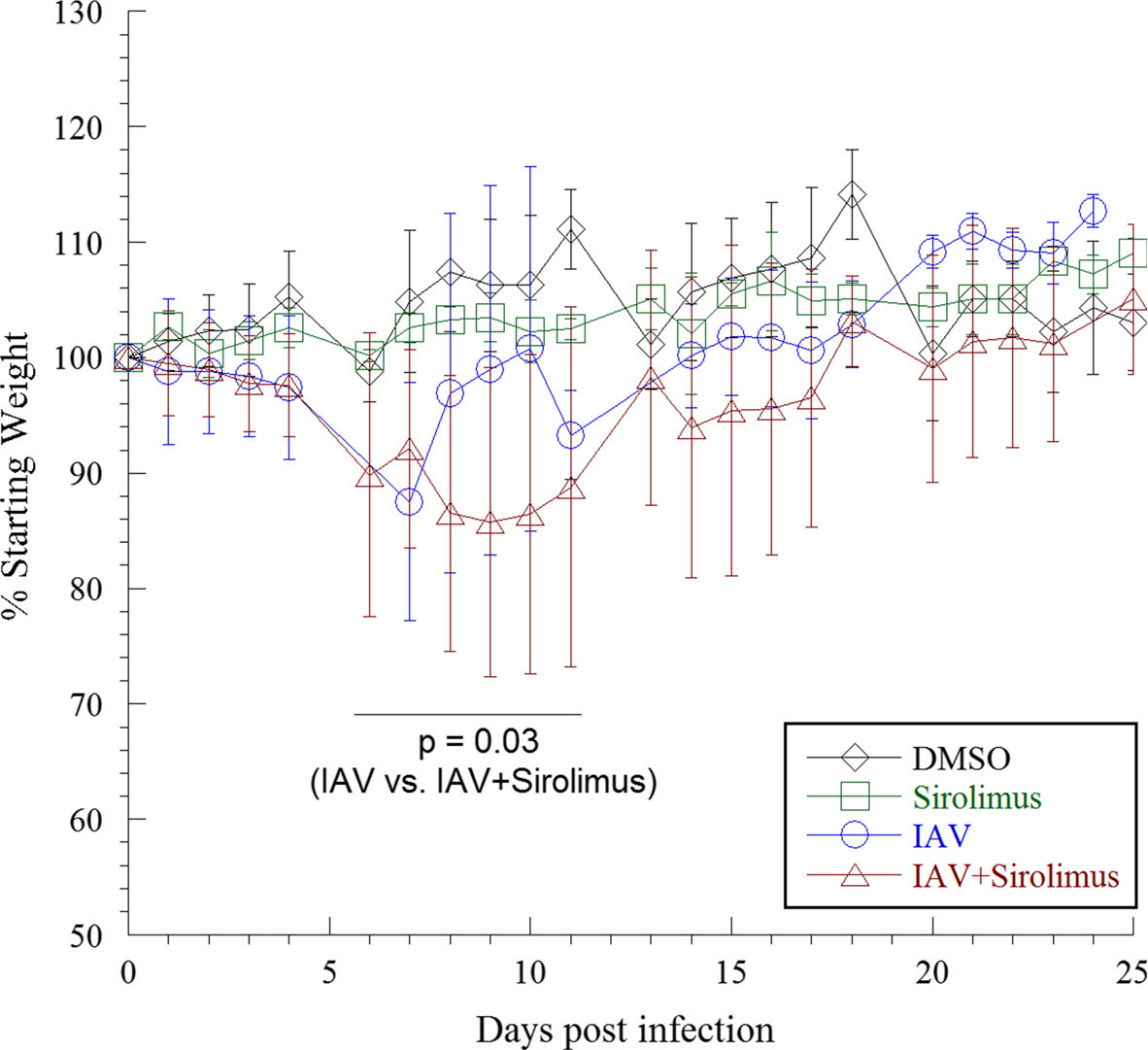
Weight changes in infected and uninfected mice. Values are mean ± SD (in percentages) of the daily weight divided by the starting weight for each mouse. Four separate experiments (9 to 20 mice per group, 56 mice) were performed. One mouse in the IAV group died on day 18, and one mouse in the sirolimus + IAV group died on day 9. The body weight loss peaked between days 6 and 11 post-infection, and was more pronounced in IAV-infected mice that were administered sirolimus than that in mice received IAV alone

The impact of sirolimus administration on the severity of IAV-induced body weight changes was monitored over 25 days. Mice were intranasally inoculated with IAV on day 0. Groups of IAV-infected mice were injected intraperitoneally with either sirolimus or an equal volume of the vehicle DMSO, both administered for 5 uninterrupted days every week for a total of 4 weeks beginning on the day of inoculation (day 0). Uninfected naïve mice administered either sirolimus or DMSO served as controls. As expected, uninfected control mice treated with either DMSO or sirolimus slowly gained weight over time. IAV-induced weight loss peaked between days 6 and 11 post-infection; averaging 12% in IAV-infected mice that were administered sirolimus (*p* = 0.002 [compared to DMSO or sirolimus alone]), in comparison to 5% in mice that received DMSO (*p* = 0.017, [compared to DMSO] or *p* = 0.030 [compared to IAV + sirolimus]), Fig. 1. Thus, the body weight loss was more pronounced in IAV-infected mice that were administered sirolimus than that in mice received IAV alone.

### Sirolimus increased the viral copies on day 10 post-infection

**Fig. 2:**
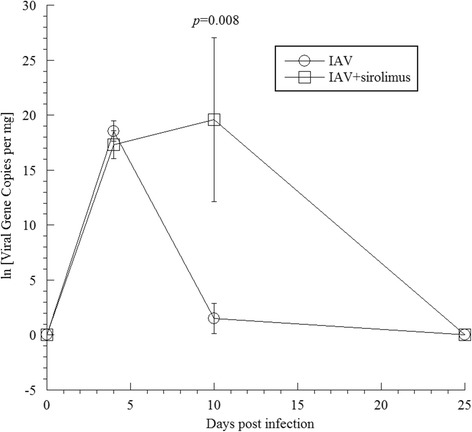
Detection of IAV in infected lungs. RNA was extracted from infected and uninfected lungs on days 4, 10, and 25, and subjected to RT-PCR using primers specific for the IAV *matrix* gene. Data represent three separate experiments (2–6 mice per group per day, 26 mice). On day 4 post-infection, the natural log viral gene copies in IAV-infected mice that were administered sirolimus were similar to those in mice received IAV alone. On day 10 post-infection, the viral gene copies were significantly higher in IAV-infected mice that were administered sirolimus than that in mice received IAV alone. Viral gene copies were not detected on day 25 in both groups

In order to determine the impact of sirolimus administration on IAV replication, we monitored viral copy numbers at selected time points following IAV infection. Figure 2 shows the viral copies on days 4, 10 and 25 post-infection. Natural log viral gene copies on day 4 in IAV-infected mice that were administered sirolimus were similar to those in mice that received IAV alone (*p* = 0.132). However, the number of copies on day 10 in IAV-infected mice that were administered sirolimus were approximately 13-fold higher than in mice that received IAV alone (*p* = 0.008). Viral gene copies were not detected on day 25 in either group.

### Sirolimus altered the lung inflammation in IAV-infected mice

**Fig. 3:**
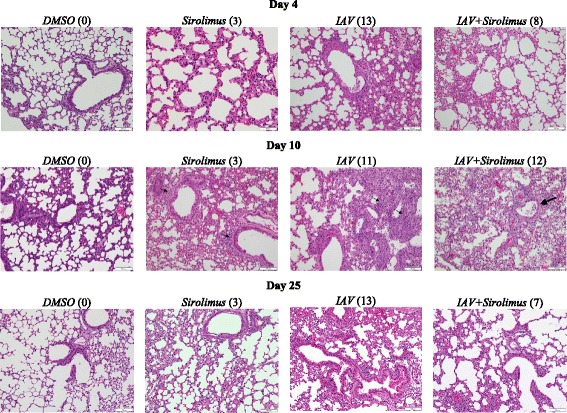
Lung histology in infected and uninfected mice. Representative H&E stained lung sections (20×) on days 4, 10 and 25 from 1 of 3 mice per condition are shown (3 separate experiments, see Table 1). *Arrows* point to areas of patchy interstitial inflammatory infiltrate with alveolar wall thickening. Numbers in parentheses represent score of inflammation. Lung histology in mice treated with sirolimus alone or IAV alone showed a slightly thickened interstitium on day 4 post-infection and peribronchial inflammation (*small arrows*) on day 10 post-infection. Peribronchial inflammation (*large arrow*) on day 10 post-infection was more prominent in IAV-infected mice that were administered sirolimus

In order to determine the impact of sirolimus administration on the severity of pulmonary disease, we monitored lung histology following IAV infection. As expected, mice that received DMSO did not exhibit any peribronchial inflammation and pulmonary parenchymal architecture was preserved. In contrast, lung histology in mice received sirolimus alone showed focal interstitial thickening and inflammation on day 4, and mild peribronchial inflammation on day 10 (Fig. 3, arrows). On days 4, 10, and 25 post-infection, lung findings in mice infected with IAV showed various degrees of patchy peribronchial and perivascular inflammation with dense, diffuse parenchymal inflammation and formation of lymphoid nodules. IAV-infected mice treated with sirolimus also showed somewhat similar patchy peribronchiolar inflammation with increased peribronchial inflammation (patchy and interrupted, most prominent on day 10 post-infection), Fig. 3. Lung inflammation reached a disease score of 9.0 ± 4.5 in IAV-infected mice that were administered sirolimus, as compared to 11.5 ± 4.5 in mice that received IAV alone (*p* = 0.335), Table 1.

### Evaluation of functional respiratory disease

**Fig. 4:**
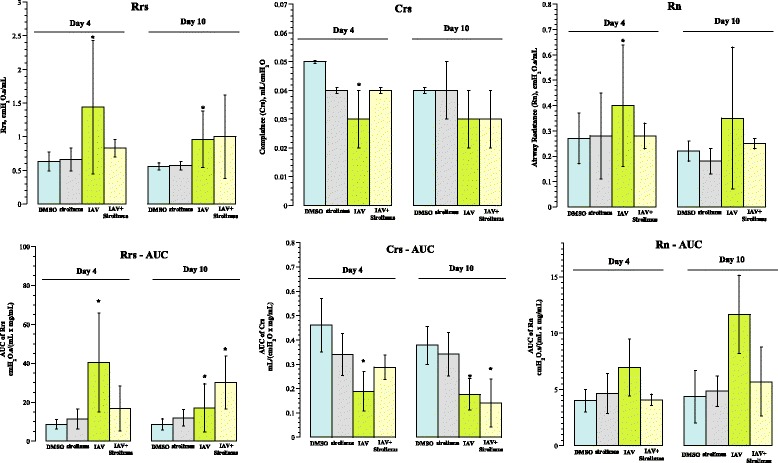
Functional respiratory assessment on days 4 and 10 post-infection. *Upper Panels*: Baseline airway reactivity expressed as thoracic resistance (Rrs), thoracic compliance (Crs), and large airway resistance (Rn). *Lower Panels*: Methacholine responsiveness shown as ‘area under the curve’ (AUC) of Rrs, Crs and Rn against methacholine concentration. The values are mean ± SD (3 to 5 separate experiments, 3 to 7 mice per condition, 33 mice). *Asterisks* designate *p* < 0.05 compared with DMSO. Increased thoracic and large airway resistances and decreased thoracic compliance were evident in IAV-infected mice on days 4 and 10 (*upper panels*). These changes (at both baseline and after methacholine) were less pronounced on day 4 and more severe on day 10 in IAV-infected mice that were administered sirolimus (*lower panels*)

In order to determine the impact of sirolimus administration on pulmonary function, we monitored lung function changes following IAV infection at selected time points post-infection. Invasive pulmonary function analysis was performed using the FlexiVent instrument, as previously described [18]. This forced oscillation system assessed the respiratory disease in tracheotomized mice by measuring thoracic resistance (Rrs), thoracic compliance (Crs), large airway resistance (Rn), lung tissue damping (resistance, G), and lung tissue elastance (H). These parameters were determined at baseline and after methacholine challenge (Fig. 4). In addition, fast-flow maneuvers, such as forced expiratory volume at 50 ms (FEV0.05) were also determined (Table 2). Impaired lung function was evident in IAV-infected mice on days 4 and 10, mainly showing increased thoracic and large airway resistances and decreased thoracic compliance. The changes (at both baseline and after methacholine) were less pronounced on day 4 and more severe on day 10 in IAV-infected mice that were administered sirolimus (Fig. 4).

**Table 2:** Respiratory functions on days 4 and 10 post-infection

		*Rrs* cmH_2_O.s/mL	*Crs* mL/cmH_2_O	*Rn* cmH_2_O.s/mL	*G* cmH_2_O/mL	*H* cmH_2_O/mL	*FEV*_*0.05*_ mL
Day 4	DMSO (*n* = 3)	0.61 ± 0.10	0.05 ± 0.0	0.27 ± 0.07	3.9 ± 0.5	19.4 ± 1.4	0.87 ± 0.18
	Sirolimus (*n* = 4)	0.66 ± 0.17	0.04 ± 0.0	0.28 ± 0.17	4.6 ± 1.1	25.0 ± 3.2	0.79 ± 0.08
	IAV (*n* = 7)	*1.44 ± 0.99* ^*^	0.03 ± 0.01*	0.40 ± 0.24	9.8 ± 6.1*	36.5 ± 18.3*	0.59 ± 0.41
	Both (*n* = 4)	0.83 ± 0.13	0.04 ± 0.0	0.28 ± 0.05	5.7 ± 1.2	22.7 ± 2.4	0.48 ± 0.39
Day 10	DMSO (*n* = 5)	0.56 ± 0.05	0.04 ± 0.00	0.22 ± 0.04	4.2 ± 0.4	20.7 ± 1.6	0.84 ± 0.24
	Sirolimus (*n* = 5)	0.57 ± 0.06	0.04 ± 0.01	0.18 ± 0.05	4.7 ± 0.6	22.4 ± 5.2	0.95 ± 0.11
	IAV (*n* = 5)	0.96 ± 0.42*	0.03 ± 0.01	0.35 ± 0.28	6.3 ± 1.5*	30.7 ± 8.3	0.53 ± 0.48
	Both (*n* = 4)	1.0 ± 0.62	0.03 ± 0.01	0.25 ± 0.02	8.8 ± 6.3	45.0 ± 34.1	0.47 ± 0.53

Rrs, thoracic resistance; Crs, thoracic compliance; Rn, large airway resistance; G, lung tissue damping (resistance); H, lung tissue elastance (stiffness); FEV 0.05, forced expiratory volume at 50 ms

Values are mean ± SD

*designates *p* < 0.05 compared with DMSO

## Discussion

Our results indicate that sirolimus administration causes more severe weight loss associated with increased viral replication. Overall, our data support the concept that mTOR signaling plays a protective role in IAV-induced lung inflammation [13]. The higher viral load on day 10 post-infection (Fig. 2) suggests a need for mTOR signaling early in the course of influenza infection. Subsequently, viral replication is apparently controlled by mechanisms that remain intact in sirolimus treated mice [13]. The early-uninhibited viral replication in the sirolimus-treated mice is responsible for the altered disease with augmented weight loss (Fig. 1). The increased viral titers at day 10 indicates that mTOR signaling is critical during the adaptive immune phase of the infection that is responsible for clearance of the virus.

The lower lung inflammatory score on day 25 post-inoculation in mice that received both IAV and sirolimus (Table 1) is consistent with a previous report showing drug-promoting apoptosis lowers experimental lung injury associated with influenza infection [19]. Similarly, using lipopolysaccharide–induced acute lung injury in mice, sirolimus has been shown to reduce inflammatory cytokines and T cell markers in the lung airway [20].

The influenza non-structural protein 1 (NS1) stimulates PI3K survival pathways and suppresses apoptosis [21]. This early event can be blocked by sirolimus, which is well known to induce thymus involution due to cortical lymphocyte apoptosis [3]. At later stages of infection, the viral protein PB1-F2 disturbs mitochondrial function and induces apoptosis [22]. The effects of sirolimus at these stages would be additive, since the drug is well known to disturb mitochondrial function and induce apoptosis [4, 5].

Inflammatory lung disease, on the other hand, is a recognized effect of mTOR inhibitors [1, 2]. We consistently observed a mild increase in respiratory dysfunction in mice that were administered sirolimus alone (Fig. 4). The primary complex of mTOR, mTORC1, imposes anti-inflammatory responses in the lung [2]. The epithelial mTORC1 has been shown to protect against endotoxin-induced acute respiratory distress syndrome and its inhibition by the endogenous inhibitor Rtp801 or by rapamycin exacerbates lung inflammation [2]. In another study, temsirolimus (converts to sirolimus in vivo) administration to mice at 2.5 μg/g resulted in alveolar epithelial injury, increased pulmonary inflammation, and induction of pro-inflammatory cytokines [1]. These results are consistent with the known potent immunosuppressive effects of this class of drugs (inducing severe thymus involution) [3].

The lung viral load on day 10 post-infection in mice that were administered sirolimus highlights the importance of immune competency and mTOR signaling in controlling lung viral load. Nevertheless, IAV was cleared from the lung on day 25 post-infection in both groups of mice. The clearance of the virus on day 25 post-infection (Fig. 2) is consistent with the recently reported preserved antibody responses to influenza infection in mice treated with sirolimus [13].

The daily sirolimus dose (5 μg/g) used here has been previously used for BALB/c mice of 10 weeks of age [14]. In one study, sirolimus administration (5 μg/g/day) induced significant weight loss in old C57BL/6 mice [23]. Further studies are needed to address the effects of administering lower sirolimus dosing on influenza infection with several virus strains.

## Conclusion

We evaluated the impact of sirolimus treatment on lung inflammation, pulmonary function and viral load following acute influenza virus infection. Mice treated with sirolimus exhibited altered lung inflammation and function, consistent with the known potent immunosuppressive effects of the drug. Sirolimus also altered lung viral load. Thus, sirolimus modified adversely the host responses to influenza infection.

The following findings support that sirolimus deteriorates IAV infection in our mouse model. Body weight loss was more pronounced in IAV-infected mice that were administered sirolimus than that in mice received IAV alone. In addition, the viral gene copies on day 10 post-infection were significantly higher in IAV-infected mice that were administered sirolimus than that in mice received IAV alone. The adverse events associated with sirolimus treatment are consistent with its potent immunosuppressive activity and, thus, preclude its use in IAV infection.
